# Tumour-specific activation of a tumour-blood transport improves the diagnostic accuracy of blood tumour markers in mice

**DOI:** 10.1016/j.ebiom.2024.105178

**Published:** 2024-06-17

**Authors:** Christian Schmithals, Bianca Kakoschky, Dominic Denk, Maike von Harten, Jan Henrik Klug, Edith Hintermann, Anne Dropmann, Eman Hamza, Anne Claire Jacomin, Jens U. Marquardt, Stefan Zeuzem, Peter Schirmacher, Eva Herrmann, Urs Christen, Thomas J. Vogl, Oliver Waidmann, Steven Dooley, Fabian Finkelmeier, Albrecht Piiper

**Affiliations:** a Goethe University Frankfurt, University Hospital, Medical Clinic 1, Frankfurt am Main, Germany; b Frankfurt Cancer Institute, Goethe University Frankfurt, University Hospital, Frankfurt am Main, Germany; c Pharmazentrum Frankfurt / ZAFES, Goethe University Frankfurt, University Hospital, Frankfurt am Main, Germany; d Molecular Hepatology-Alcohol Associated Diseases, Department of Medicine II, Medical Faculty Mannheim, University of Heidelberg, Germany; e Suez University, Faculty of Science, Zoology Department, Suez, Egypt; f Institute of Biochemistry II, Faculty of Medicine, Goethe University, Frankfurt am Main, Germany; g Department of Medicine I, University Medical Centre Schleswig-Holstein - Campus Lübeck, Lübeck, Germany; h German Cancer Consortium (DKTK), Partner Site Frankfurt/M., a Partnership Between DKFZ and University Hospital Frankfurt/M., Germany; i Institute of Pathology, University of Heidelberg, Germany; j Goethe University Frankfurt, University Hospital, Institute of Biostatistics and Mathematical Modelling, Germany; k Goethe University Frankfurt, University Hospital, Institute for Diagnostic and Interventional Radiology, Germany; l Centrum für Hämatologie und Onkologie Bethanien, Frankfurt/Main, Germany

**Keywords:** HCC, Tumour marker, α fetoprotein, iRGD, CEND-1, Early cancer detection

## Abstract

**Background:**

The accuracy of blood-based early tumour recognition is compromised by signal production at non-tumoral sites, low amount of signal produced by small tumours, and variable tumour production. Here we examined whether tumour-specific enhancement of vascular permeability by the particular tumour homing peptide, iRGD, which carries dual function of binding to integrin receptors overexpressed in the tumour vasculature and is known to promote extravasation via neuropilin-1 receptor upon site-specific cleavage, might be useful to improve blood-based tumour detection by inducing a yet unrecognised vice versa tumour-to-blood transport.

**Methods:**

To detect an iRGD-induced tumour-to-blood transport, we examined the effect of intravenously injected iRGD on blood levels of α-fetoprotein (AFP) and autotaxin in several mouse models of hepatocellular carcinoma (HCC) or in mice with chronic liver injury without HCC, and on prostate-specific antigen (PSA) levels in mice with prostate cancer.

**Findings:**

Intravenously injected iRGD rapidly and robustly elevated the blood levels of AFP in several mouse models of HCC, but not in mice with chronic liver injury. The effect was primarily seen in mice with small tumours and normal basal blood AFP levels, was attenuated by an anti-neuropilin-1 antibody, and depended on the concentration gradient between tumour and blood. iRGD treatment was also able to increase blood levels of autotaxin in HCC mice, and of PSA in mice with prostate cancer.

**Interpretation:**

We conclude that iRGD induces a tumour-to-blood transport in a tumour-specific fashion that has potential of improving diagnosis of early stage cancer.

**Funding:**

10.13039/501100005972Deutsche Krebshilfe, 10.13039/501100012353DKTK, LOEWE-Frankfurt Cancer Institute.

**Research in context**

**Evidence before this study**

The diagnosis of cancer at early stages is crucial to apply potentially curative therapeutic options. Blood tumour markers would be optimal to achieve this. However, the accuracy of blood-based tumour markers is compromised by simultaneous production at non-tumoral sites, individual variant expression in the tumours and too low production by small tumours to elicit detectable changes in the blood. The particular tumour homing peptide iRGD is known to specifically target tumour vessels via its RGD motif and subsequently to induce extravasation of co-administered substances specifically in tumours through binding to neuropilin-1 receptor. Preliminary evidence suggests that this also holds for patients. Whether the iRGD-induced transport in the tumour vessels may also act in the opposite direction and induce a diagnostically useful transport of tumour markers, leading to detectable changes in their blood concentrations, was unknown.

**Added value of this study**

Examination of the blood levels of tumour-derived tumour marker α-fetoprotein (AFP) before and after administration of iRGD in several mouse models of HCC and chronic liver disease revealed that iRGD induced a rapid onset tumour-blood transport in mice with hepatocellular carcinoma (HCC). iRGD had no effect on mice with non-tumoral AFP production due to chronic liver disease. The iRGD-induced tumour-to-blood transport of AFP was found to depend on neuropilin-1 and on the concentration gradient between the tumour and the blood, and was primarily seen in animals with small tumours and basally normal blood AFP levels. iRGD also induced tumour-blood transport of the secretory protein autotaxin in HCC mice, and of prostate-specific antigen (PSA) in mice with prostate cancer, suggesting that iRGD-induced transport may not be confined to classical tumour markers and extend beyond liver cancer.

**Implications of all the available evidence**

iRGD causes a transport of tumour-derived substances from the tumour into the blood in a tumour-specific fashion. It has potential of improving the diagnostic accuracy of diverse tumour-released blood markers at early stages of HCC and potentially other cancers.

## Introduction

For the majority of cancers, early detection enables the application of potentially curative treatments, leading to improved patient survival. For instance, in liver cancer, the third leading cause of cancer-related mortality worldwide, among which hepatocellular carcinoma (HCC) accounts for the majority of cases, the 5-year survival rate exceeds 70% for patients with early-stage HCC, but survival is 1–1.5 years for advanced-stage HCC.^1^, ^2^, ^3^ Unfortunately, due to a lack of methods for early diagnosis, a majority of patients with cancer is diagnosed at an advanced stage with limited therapeutic options and thus poor prognosis. Blood markers that detect malignant tumours at an early stage with high sensitivity and specificity could improve this situation. The tumour markers currently in clinical use, such as α-fetoprotein (AFP) in HCC and prostate-specific antigen (PSA) in prostate cancer, show insufficient sensitivity and specificity to be effective in early diagnosis of the cancer.^4^, ^5^, ^6^, ^7^, ^8^ Emerging tumour markers such as circulating tumour DNA (ctDNA) or cell-free RNA, hold promise to enable more accurate early tumour diagnosis in the future.^7^, ^8^, ^9^, ^10^ Major limitations of the early detection of tumours by tumour-released markers are elevated blood levels from non-malignant conditions (e. g. inflammation), individual variation of tumour expression, and undetectable amounts released by small tumours.^11^, ^12^, ^13^

In order to reach the blood circulation, tumour markers must be transported from the tumour microenvironment across the tumour vascular endothelium into the vascular lumen. Recently, a particular nine amino acid circular peptide, termed internalizing (i)RGD, has been discovered that selectively increases the permeability of tumour vessels in diverse mouse tumour models upon its intravenous injection, as revealed by increased transport of diverse kinds of substances specifically into the tumours upon co-injection of iRGD.^14^, ^15^, ^16^, ^17^, ^18^, ^19^ iRGD contains an RGD motif, an internal C- end Rule (CendR) motif (R/KXXR/K), and a proteolytic cleavage recognition site. Intravenously injected iRGD homes to tumours by initially binding to αvβ3 and αvβ5 integrins that are selectively expressed on tumour blood vessels,^20^ via its RGD motif.^21^ It is then proteolytically cleaved to produce a peptide with C-terminal exposed CendR motif, which loses affinity for integrins and binds to neuropilin-1 (NRP-1), a cell surface receptor that plays a fundamental role in mediating vascular permeability signals.^22^^,^^23^ NRP-1 plays an essential role in iRGD-induced extravasation in tumours,^15^ and CendR peptide-induced vascular permeability.^24^^,^^25^ The permeability route induced by CendR peptide stimulation may involve paracellular or transcellular pathways, or both.^23^ iRGD induces the formation of grape-like vesicular structures in the tumour vascular endothelium of pancreatic and gastric cancer in mice,^26^^,^^27^ and silicasomes co-administered with iRGD were found within these vesicles,^26^ suggesting that these vesicular structures mediate the iRGD-induced transvascular transport. The iRGD-induced vesicular structures resemble the vesicular structures induced by vascular endothelial growth factor-A in vascular endothelia, termed vesiculo-vacuolar organelles (VVOs), which can fuse and appear as channels crossing single cells and transport protein-sized or larger proteins.^28^^,^^29^ It is also possible that iRGD induces paracellular permeability of the vascular epithelium.^30^

Here we investigated whether the NRP-1-dependent transvascular transport activated by iRGD may also act in the opposite direction, i. e. as tumour-blood transport, when the concentration gradient of the substance to be transported favours this. The data of the present study indicate that iRGD induced a tumour-blood transport as detected by a rapid elevation of the blood AFP level in mice with HCC. This transport was tumour-specific and occurred preferentially in animals with small HCCs and low basal AFP levels. This may have potential of improving cancer diagnostics in HCC and other cancers at early tumour stages.

## Methods

### Peptides

iRGD (CRGDKGPDC) and RGD control peptide (CRGDDGPKC) were synthesised by GenScript ([Piscataway, NJ], USA) as cyclic peptides with a disulphide bond between amino acids 1 and 9. CendR peptide (CRGDK) was also from GenScript. The reported purity was higher than 98%.

### Ethics

All animal experiments have been approved by the local Ethics Animal Review Broad Hessen (Darmstadt, Germany, approval numbers F34/10, FK/1100, FK/1139 and FK/2006) were carried out in accordance with the recommendations of the Animal Protection Agency of the Federal State of Hessen (Regierungspräsidium Darmstadt, Germany). All of mice were fed and housed in the central animal research facility of the University Hospital Frankfurt. The investigators carried out the animal experiments were not blinded to the groups. All mice used in the study were age-matched and exclusively male, because of their higher susceptibility to develop HCC as compared to female mice.

### Nude mice experiments

HepG2 (RRID:CVCL_0027) and Huh-7 (RRID:CVCL_0336) cells were obtained from ATCC (Manassas, VA) and RIKEN BioResource Centre (Ibaraki, Japan), respectively. LNCaP cells (RRID:CVCL_0395) were obtained from the Leibnitz Institute DSMZ (Braunschweig, Germany). All cell lines were grown in DMEM supplemented with 10% FBS and penicillin/streptomycin (Life Technologies, Waltham, MA), and were routinely monitored for morphologic and growth characteristics and mycoplasma. Early passages following authentication were used.^31^ A total of 1–10 × 10^6^ cells (suspended in 100 μl of PBS) were injected subcutaneously into the flanks of female (HepG2, Huh-7) or male (LNCaP) NMRI Foxn1 nude mice (RRID:IMSR_ENV:HSD-889, Envigo, Huntingdon, UK).

### Mouse models of liver fibrosis

An autoimmune model of liver fibrosis was generated by injecting 6 weeks old male FVB/NHsd mice (RRID:IMSR_ENV:HSD-118, Envigo) with 2 × 10^8^ pfu of Ad-2D6 i.p and i.v.^32^ Virus titres were determined with the Adeno-X rapid titre kit (Clontech, Palo Alto, CA). The mice were used for the experiments four weeks after the Ad-2D6 injection.

For carbon tetrachloride (CCl_4_, #319961 Sigma–Aldrich/Merck, Darmstadt, Germany)-induced liver fibrosis, male FVB mice (RRID:MGI:3609372) aged 6–8 weeks were treated twice weekly by intraperitoneal injection of 5 μl CCl_4_ diluted 1:20 in corn oil for four weeks.^33^ The mice were used for the experiments on the day following the last CCl_4_ injection. As a third model of liver fibrosis, six months old Mdr2^−/−^ mice (RRID:IMSR_JAX:002539) in FBV background were used.^34^

### Generation of transgenic mice and visualisation of HCC

The animals were inspected every 2–3 days. Male transforming growth factor-α (TGFα)/c-myc mice were generated by crossing homozygous metallothionein/TGFα and albumin/c-myc mice in CD13B6CBA background (kindly provided by Snorri Thorgeirsson (National Cancer Institute, NIH, Bethesda) as described.^35^^,^^36^ After weaning, hepatocarcinogenesis in the mice was accelerated by ZnCl_2_ in the drinking water. The endogenously formed HCCs were detected and monitored by gadoxetic acid (Gd-EOB-DTPA, #RR-PRIM-DE-0178-1, Bayer, Leverkusen, Germany)-enhanced magnetic resonance imaging (MRI) in a 3T MRI scanner (Siemens Magnetom Trio, Siemens Medical Solution Health Service, Eschborn, Germany) as described recently (Supplementary Fig. S1).^36^ The diameters of the tumours were measured using the program Centricity RIS 4.1i Plus, version 4.1 (GE Healthcare, Little Chalfont, UK).

### Diethylnitrosamine (DEN)/CCl_4_-induced HCC mouse model

Five weeks old male BL/6 mice (RRID:MGI:7264769) received a single injection of a low dose of DEN (1 mg/kg i. p., #N0756, Merck, Darmstadt, Germany), followed by the injection of CCl_4_ (0.2 ml/kg, in an identical volume of corn oil, i. p.) from the age of 8 weeks twice per week for 14 weeks. The endogenously formed HCCs were detected and monitored by Gd-EOB-DTPA-enhanced MRI in a 3T MRI scanner.

### Treatment of the mice with peptides and use of anti-NRP-1 blocking antibody

The mice were assigned to the different treatment groups according to age and tumour size and received iRGD, RGD control peptide (4 μmol/kg each), or PBS by tail vein injection. Blood was drawn from the mice 5 min before and 90 min after the i. v. injection of peptides/vehicle, if not stated otherwise. AFP levels were determined in the blood samples.

For neutralisation of NRP-1 in the HCC mice, TGFα/c-myc mice with HCC according to Gd-EOB-DTPA-enhanced MRI and robust iRGD-induced increase in the blood AFP concentration one week before were injected intravenously with 50 μg of a neutralising anti-mouse NRP-1 (#MAB59941, R&D Systems, Minneapolis, MN). One h later blood was drawn, followed by the injection of iRGD (4 μmol/kg) and a second blood draw after another 90 min. AFP levels were determined in sera from blood samples.

### Measurement of iRGD-induced vascular permeability

TGFα/c-myc mice with liver tumours according to Gd-EOB-DTPA–enhanced MRI received iRGD or RGD control peptide (4 mmol/kg each) by tail vein injection. Fifteen min later, Evans Blue (33.3 mg/kg, #02151108-CF, MP Biomedicals) was injected intravenously. Another 30 min later, the mice were terminally perfused with Ringer solution by cannulation of the left heart ventricle. After laparotomy, the liver, tumour, and other organs were excised and Evans Blue was extracted and measured as described.^15^^,^^17^

### ELISAs, serum ALT, AST and LDH measurements

To quantify AFP and autotaxin, 20–50 μl of blood was collected from the tail vein in an Eppendorf tube and left at room temperature for 30 min, followed by centrifugation at 1000 *g* for 5 min. The supernatant was collected in a PCR tube, re-centrifuged and stored at −20 °C until utilization for the assays. For the measurements, the sera were diluted appropriately with PBS. Quantikine ELISAs were used to detect human autotaxin (#DENP20, R&D Systems, Minneapolis, MN)^37^ as well as either mouse or human AFP were from R&D Systems (#MAFP00 and #DAFP00). The levels of total PSA were measured using the Elecsys total PSA immunoassay (#04641655190, Roche Diagnostics, Mannheim, Germany) by the Central Laboratory of the University Hospital Frankfurt. Each serum sample was analysed in duplicate. Each data point represents the mean of the duplicate. There was no background from the mouse sera in the assays specific for the human substrates.

Alanine aminotransferase (ALT), aspartate aminotransferase (AST) and lactate dehydrogenase (LDH) levels in sera were measured by the Central Laboratory of the University Hospital Frankfurt.

### Haematoxylin and eosin staining

Paraffin embedded liver sections were deparaffinised and rehydrated before incubating them in a haematoxylin bath for 8 min. The sections were washed in warm tap water for 10 min, rinsed in deionized water and 95% ethanol and counterstained in Eosin G/Y solution for 60 s. The sections were then dehydrated in 95% ethanol and pure ethanol, cleared in xylene and mounted.

### Sirius Red staining

Paraffin embedded liver sections were deparaffinised and rehydrated before incubating the tissue with 100 ml Sirius Red solution (#26357, Electron Microscopy Science, Hatfield, PA) at room temperature for 1 h. Afterwards, slides were washed with 0.01 N HCl and H_2_O, dehydrated in ethanol, cleared in xylene and mounted.

### Immunoblotting

Immunoblotting of lysates obtained from pairs of liver and HCC tissue from TGFα/c-myc mice was performed as described previously.^38^ Gel-resolved proteins were electrotransferred to nitrocellulose membranes and incubated with antibodies raised against mouse-AFP (#AF5369, R&D Systems, Minneapolis, MN, RRID:AB_2258018) and anti-β-actin (#A2066, Sigma–Aldrich/Merck, RRID:AB_476693). Antigen-antibody complexes were visualized using appropriate horseradish peroxidase-conjugated antibodies and the enhanced chemiluminescence system (Millipore, Burlington, MA). Band densities were measured densitometrically and quantified using Image J.

### Release of AFP from isolated HepG2 cells

10^5^ HepG2 cells were cultured for two days in a six-well plate in DMEM with 10% FBS and 1% penicillin/streptomycin. After two days the cells were washed twice with pre-warmed PBS and incubated in FBS-free DMEM for 1 h. Thereafter, 50 μl aliquots of the supernatants were removed (time zero), and 10 μM iRGD, 10 μM CendR peptide, 10 μM RGD control peptide or an appropriate volume of PBS were added to the cells. Additional aliquots of 50 μl of the supernatants were collected after further incubation for 30 and 90 min. All aliquots were briefly centrifuged and frozen at −80 °C until determination of the AFP concentration by ELISA (#DAPF00, R&D Systems).

### Statistics

Data are described by medians or geometric means ± 95% confidence interval (CI) or means ± SD, as indicated. All data points are from different individuals. Animals were randomly assigned to treatment groups. Exclusion criteria of experimental data were technical failure at analysis. The number of mice included in the experiments was chosen to allow subgroup analyses. Statistical analyses were performed using GraphPad Prism 10.1.1 software (GraphPad, San Diego, CA). The tests used for the analyses are described in the figure legends. Testing for normal distribution of the data was performed using the Shapiro–Wilk test and Q–Q plots. For log-normally distributed data, paired or unpaired t test or one sample t test was used for log-transformed data. In these cases, estimates of the ratio of geometric mean (fold change) and 95% CI are given. Comparisons between non-normally distributed data were performed using the Mann–Whitney U test for unpaired data. In case of paired data or one-sample comparisons, Wilcoxon matched-pairs signed-rank test or one-sample Wilcoxon signed-rank test were used for log-transformed data (after checking for symmetry). In these cases, effect sizes are given as median of the ratios with 95% CI. Multiple comparisons of non-normally distributed data were performed using the Kruskal–Wallis test with Dunn’s multiple comparison post-hoc tests and with one-way analysis of variance (ANOVA) for log-transformed data, again with multiple comparison post-hoc tests. The correlation coefficient r between different variables was calculated using Spearman correlation or Pearson correlation for log-transformed data with two-tailed p values and 95% CI.

### Role of funders

The funders played no role in the design of the research, collection, analysis and interpretation of data, the writing of the paper or the decision to submit the manuscript for publication.

## Results

### Intravenously injected iRGD leads to a rapid increase in the blood AFP concentration in mice with HCC

We reasoned that the iRGD-induced vascular permeability could also lead to the transport of substances from the tumour interstitium into the blood if the concentration gradient favours it. This transport could lead to transiently elevated levels of tumour-derived proteins such as tumour markers in the blood circulation. As iRGD induces a rapid (within minutes) activation of a transvascular transport, which may fade due to the short circulation half-life of iRGD,^39^ we expected that an iRGD-induced tumour-to-blood transport should result in a rapid increase in the blood concentration of tumour derived proteins. The clinical HCC marker AFP is a protein expressed and secreted by approximately one-third of human HCCs,^40^^,^^41^ leading to elevated AFP levels in the blood of affected patients.^4^, ^5^, ^6^, ^7^ The blood half-life of AFP is several days in humans. Thus, even a short iRGD-induced spike in AFP transport from the tumour would be expected to cause elevated blood AFP levels for at least several hours, rendering it suitable to detect a potential iRGD-induced tumour-to-blood transport.

**Fig. 1:**
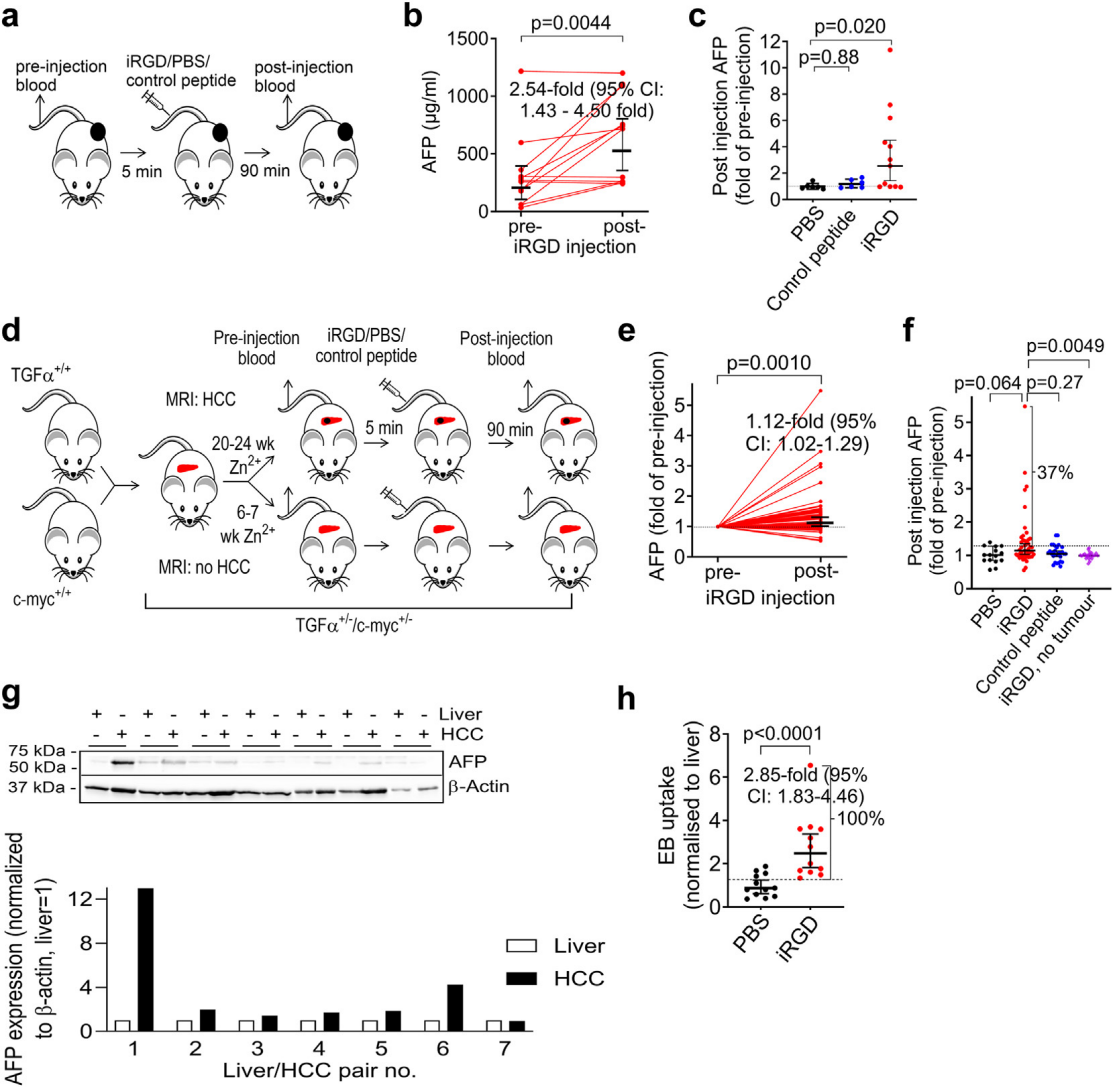
Intravenously injected iRGD increases blood AFP levels in HCC-bearing mice. **(a)** Experimental setup to study the effect of iRGD on blood AFP levels in Huh-7 xenografted nude mice. **(b and c)** Blood AFP levels before and after intravenous injection of iRGD (**b, c**, n = 12), RGD control peptide (**c** (n = 6)), and PBS (**c** (n = 6)) in mice with HepG2 xenografts. Human AFP was not detectable in the blood of mice without tumours. In **(c)** the fold increase of AFP with pre-injection level set to 1; lines and error bars represent geometric means and 95% CI. **(d)** Experimental setup to study the effect of iRGD on blood AFP levels in TGFα/c-myc HCC mice. **(e and f)** iRGD specifically increased the blood AFP levels in TGFα/c-myc HCC mice. TGFα/c-myc mice (20–24 weeks old) with HCC according to MRI or without HCC **(f)** were intravenously injected with iRGD **(e, f)**, RGD control peptide **(f)** or PBS **(f)**. Data are fold changes of blood AFP due to the treatments (n = 48: iRGD; RGD control peptide: n = 34; PBS: n = 15); lines and error bars indicate medians and 95% CI. Dashed line: upper 95% CI increase in blood AFP in the PBS-injected HCC mice. **(g)** AFP expression in HCCs of TGFα/c-myc mice. HCCs and liver tissues were excised from TGFα/c-myc mice. Pairs of the tissue lysates were analysed for AFP and β-actin content by immunoblotting. Band densities were measured densitometrically. The ratio of AFP/β-actin in the livers was set to 1. **(h)** iRGD-induced accumulation of Evans blue in HCCs in TGFα/c-myc mice with HCC. TGFα/c-myc mice with HCCs were co-injected with iRGD or PBS (n = 12 per group) and Evans blue (EB). The dye content of the tumours was related to that in the livers; lines and error bars indicate geometric means **(b, c, and h)** or medians **(e and f)** and 95% CI; dashed line: upper 95% CI of the measured EB content of a HCC from the PBS-injected animals. **(b)**: paired t test for log-transformed data; **(c)** One-way ANOVA with multiple comparison post-hoc test for log-transformed data; **(e)**: Wilcoxon signed-rank test for log-transformed data; **(f)** Kruskal–Wallis test with Dunn’s multiple comparison post-hoc test; **(h)**: two-sample t test. The indicated fold increase in **(b, e and h)** is the ratio of the geometric means with 95% CI.

We injected nude mice bearing AFP-expressing HCC xenografts, which show iRGD-induced tumour uptake,^17^ with iRGD, a RGD control peptide or PBS, and analysed blood samples drawn 5 min before and 90 min after the injections for AFP concentrations (Fig. 1a). The relatively early point of time at 90 min after peptide/control injections was chosen to minimize the risk of alterations of the blood AFP level by iRGD-independent events (e. g. tumour progression). iRGD appeared to cause an increase of the blood AFP levels in mice with Huh-7 (Fig. 1b and c) or HepG2 tumours (Supplementary Fig. S2a and b), whereas there was no evidence that the injection of an RGD control peptide lacking the CendR motif or PBS affected the blood AFP levels (Fig. 1c, Supplementary Fig. S2c). These data suggest that iRGD caused an elevation of the blood AFP level in HCC mice, which required the CendR motif, and that the effect of iRGD was not simply a consequence of the blood drawing.

There was no indication that iRGD increased the levels of alanine aminotransferase (ALT), aspartate aminotransferase (AST) and lactate dehydrogenase (LDH) in HCC xenografted mice (Supplementary Fig. S2d), suggesting that cell damage was not involved in the iRGD-induced elevation of circulating AFP in these mice.

To investigate whether the effect of iRGD on the AFP blood level can be detected in mice with endogenously formed HCCs and can be discriminated from the background AFP produced by non-tumoral sites, we examined the effect of iRGD on the blood AFP level in conditional double transgenic TGFα/c-myc mice with HCC (Fig. 1d). These mice develop HCCs upon induction of transgene expression by zinc in the drinking water.^35^^,^^36^ Measurement of the AFP levels before and after intravenous injection of iRGD into TGFα/c-myc mice with HCC according to contrast-enhanced MRI were compatible with an iRGD-induced increase of the blood AFP level in these mice (Fig. 1e). However, only approximately a third of these HCC mice injected with iRGD showed a higher increase of the blood AFP level than all the HCC mice injected with PBS (Fig. 1f), complicating the investigation of iRGD-induced tumour-blood transport in this HCC mouse model. No alterations of the blood AFP levels were observed in HCC mice injected with RGD control peptide or PBS (Fig. 1f), or in 6–7 weeks old TGFα/c-myc mice injected with iRGD, i. e. prior to tumour development (Fig. 1f). As only mice with AFP expressing HCCs can show an iRGD-induced tumour-to-blood transport of AFP, we examined the levels of APF in tumours and corresponding livers of TGFα/c-myc HCC mice by immunoblotting. Approximately 40% of the tumours showed higher AFP expression than the livers of the same animal (Fig. 1g), which may explain the only partial response rate of the blood AFP levels to the injection of iRGD in these mice. On the other hand, iRGD caused an extravasation of co-injected albumin-binding dye Evans blue in the majority of HCCs (Fig. 1h), a robust method to detect vascular integrity,^42^ indicating that the majority of these HCC mice showed nevertheless an iRGD-inducible tumour vascular transport.

**Fig. 2:**
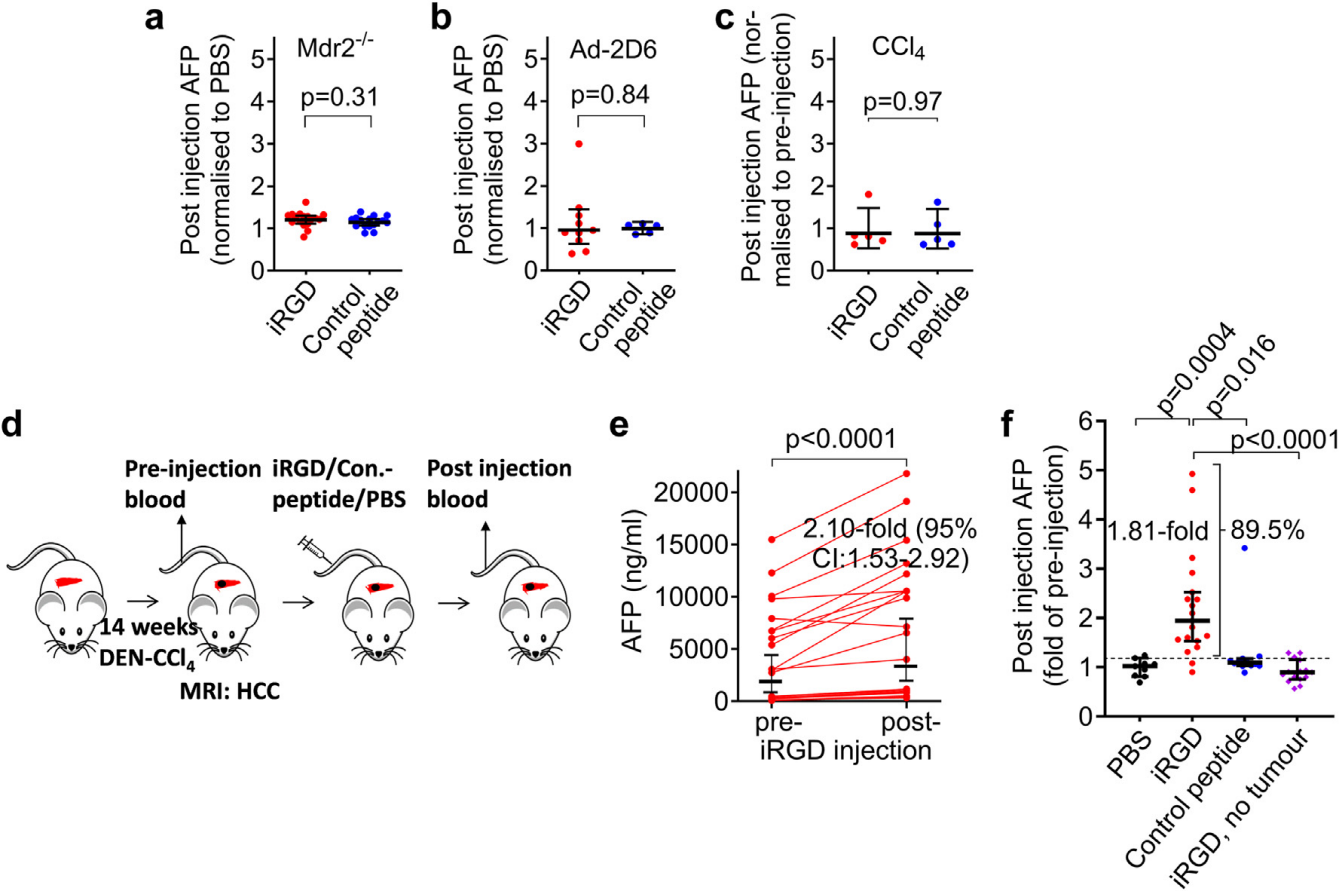
iRGD increases the blood AFP concentration in mice with HCCs formed endogenously in fibrotic livers, but not in mice with liver fibrosis only. **(a–c)** iRGD had no effect on blood AFP in mice with liver fibrosis. Six-month-old Mdr2^−/−^ mice (**a**, n = 16–17) as well as mice treated with Ad-2D6 (**b**, n = 5–10) or CCl_4_ (**c**, n = 3–5) for four weeks were bled before and after the injection of iRGD or RGD control peptide and was analysed for AFP content. **(d–f)** iRGD increased blood AFP in mice with liver fibrosis and HCC. **(d)** Experimental setup. **(e and f)** Mice with HCC were injected intravenously with iRGD (n = 19), RGD control peptide (n = 12), PBS (n = 9), or the mice were injected with iRGD prior to tumour development (n = 23). For the latter, mice treated for 12 weeks with DEN-CCl_4_ received contrast-enhanced MRI to show absence of liver tumours at this time of induction of hepatocarcinogenesis; **(f)** fold increase of AFP with pre-injection level set to 1; dashed line: upper 95% CI increase in blood AFP in the PBS-, RGD control peptide-treated HCC animals and in iRGD-treated animals without tumour. **(a–c, e, f)**: Lines and error bars represent geometric means **(a–c)** or medians **(e and f)** with 95% CI. **(a–c)**: Unpaired t test for log-transformed data with Welch correction **(b)**; **(e)**: Wilcoxon matched-pairs signed-rank test; **(f)**: Kruskal–Wallis test with Dunn’s multiple comparison post-hoc test. The indicated fold increase in **(e)** is the median of the ratios.

To investigate whether the proposed iRGD-induced tumour-to-blood transport of AFP is indeed tumour-specific or also occurs in mice with chronic hepatic inflammation and non-tumoral AFP production, we examined the effect of iRGD on blood AFP levels in three different mouse models of liver fibrosis (Ad-2D6 virus-induced autoimmune hepatitis, Mdr2^−/−^ mice or CCl_4_ treatment). Ad-2D6 and CCl_4_-treated mice with histologically confirmed liver fibrosis (Supplementary Fig. S3a) presented with elevated blood AFP levels (Supplementary Fig. S3b and c), whereas the Mdr2^−/−^ mice had normal AFP levels. The data obtained in these mice were compatible with a lack of effect of iRGD treatment on the blood AFP levels in the three mouse models of liver fibrosis (Fig. 2a–c), suggesting that iRGD may not affect AFP levels in mice with fibrotic livers in the absence of HCC.

To examine if the iRGD-induced transport of AFP is detectable in mice with endogenously formed HCCs in the background of liver fibrosis, we examined the effect of iRGD in DEN-CCl_4_-treated mice with radiologically visualized HCCs (Fig. 2d). This model combines chronic liver injury, inflammation, and fibrogenesis with hepatocarcinogenesis, thus sharing several features with the majority of human HCCs.^43^ Blood AFP levels were already elevated after 12 weeks of DEN-CCl_4_-treatment (283 ± 198.4 ng/ml [mean ± SD, n = 8]) in animals without tumour, indicating AFP production by chronic liver damage. In mice with DEN-CCl_4_-induced HCCs on a fibrotic background (Supplementary Fig. S4), the measured blood AFP levels before and after the injection of iRGD were compatible with an iRGD-induced increase of the blood AFP level (Fig. 2e), whereas RGD control peptide or PBS had no measurable effect (Fig. 2f). In ∼90% of HCC mice, iRGD led to a relative increase of the blood AFP concentration by at least the upper 95% CI increase in blood AFP level in the control animals (Fig. 2f). When iRGD was intravenously injected into mice treated with DEN-CCl_4_ prior to tumour development (week 15–19), iRGD had no effect on blood AFP levels (Fig. 2f). This suggests that iRGD induced an elevation of the blood AFP level in animals with HCC in fibrotic livers and that this effect depended on the presence of HCC.

### iRGD-induced elevation of the blood AFP level depends on NRP-1 and the tumour-blood concentration gradient of AFP

The iRGD-induced increase in the blood AFP concentration in HCC mice could be due to the stimulation of AFP secretion from the tumour cells rather than an iRGD-induced tumour-to-blood transport of AFP. Therefore, we investigated the effect of iRGD, its potential cleavage product CendR peptide that directly interacts with NRP-1,^24^ and of RGD control peptide on AFP release from cultured HepG2 cells. The data were compatible with no effect of any of the peptides on AFP release from cultured HepG2 cells (Supplementary Fig. S5), suggesting that iRGD did not affect constitutive secretion of AFP from HCC cells.

**Fig. 3:**
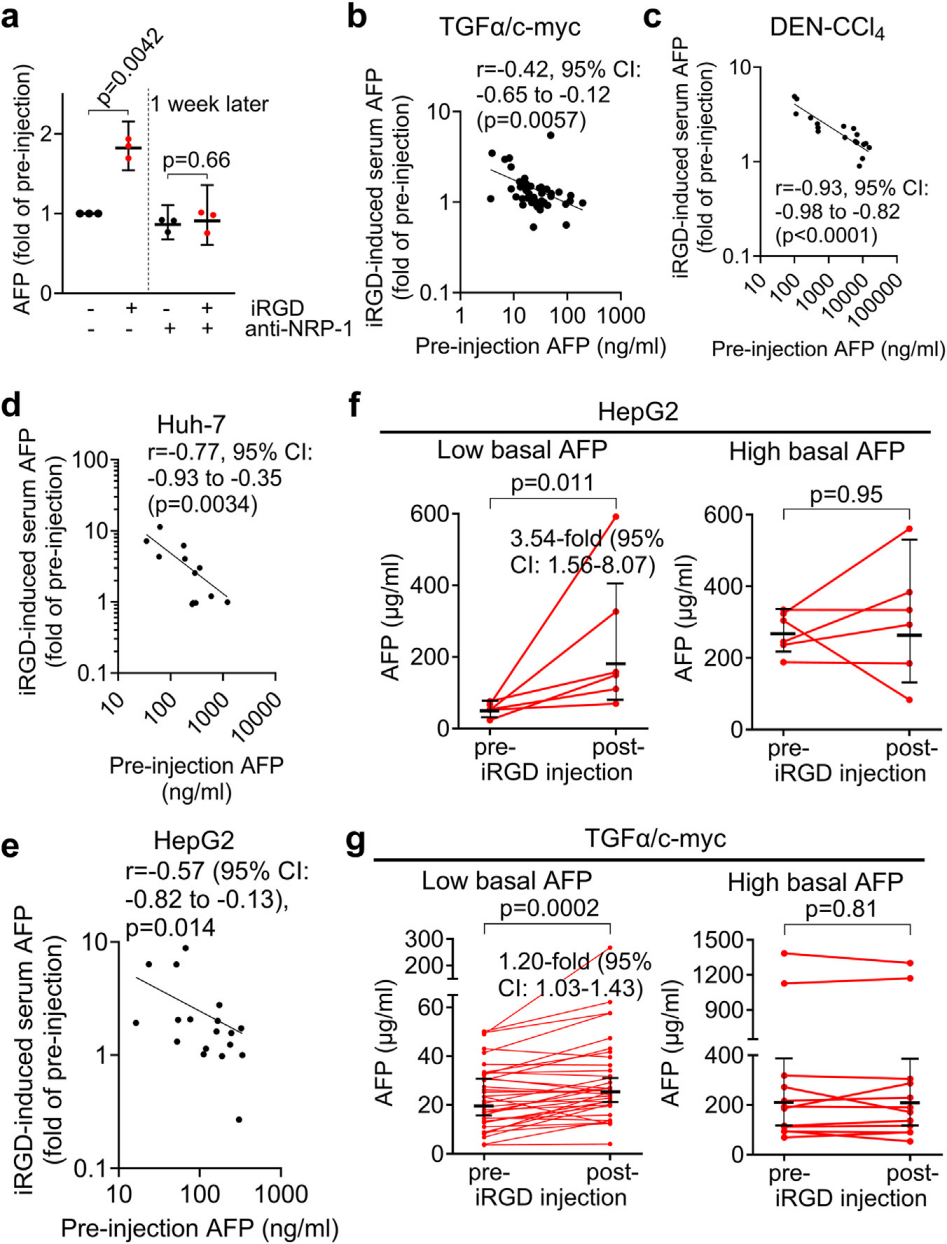
iRGD-induced elevation of the blood AFP concentration depends on NRP-1 and the tumour blood concentration gradient of AFP. **(a)** Anti-NRP-1 prevented iRGD-induced increase in the blood AFP concentration. TGFα/c-myc tumour mice that displayed a robust iRGD-induced increase in blood AFP level one week earlier were injected with anti-NRP-1 and the effect of iRGD on blood AFP level was determined (n = 3 per group). Fold increase of AFP with pre-injection level at the first time point was set to 1. Lines and error bars represent medians and 95% CI. **(b–e)** iRGD-induced elevation of the blood AFP level correlated negatively with the pre-injection blood AFP level in TGFα/c-myc mice **(b)**, DEN-CCl_4_-HCC mice **(c)**, mice with Huh-7 **(d)** or HepG2 xenografts **(e)**. Spearman correlation r **(b and c)** and Pearson correlation r **(d and e)** with 95% CI, two tailed p-values and the log–log regression lines. **(f)** iRGD increased the blood AFP levels in HepG2 xenografted nude mice and low basal AFP (<67 ng/ml, n = 36, left panel), but not in animals with high basal AFP (>67 ng/ml, n = 12, right panel). Lines and error bars indicate geometric means and 95% CI. [**(g)** iRGD increased the blood AFP levels in TGFα/c-myc mice with HCC and normal basal AFP (<67 ng/ml, n = 36, left panel), but not in mice with elevated basal AFP (>67 ng, n = 12, right panel). Lines and error bars represent medians (left) or geometric means (right) with 95% CI. Significance was calculated with one sample t test (**a**, left) and the unpaired t test (**a**, right), paired t test (**f and g**, right) and Wilcoxon matched-pairs signed-rank test (**g**, left). The indicated fold increase in **(f)** is the geometric mean ratio with 95% CI. The indicated fold increase in **(g)** is the median of the ratios with 95% CI.

The iRGD-induced vascular permeability and tumour import in pancreatic cancer strictly depend on NRP-1.^15^^,^^24^ To investigate if the iRGD-induced transport of AFP depends on NRP-1 as well, we studied the effect of a neutralizing anti-NRP-1 antibody on iRGD-induced increase of the blood AFP level in the TGFα/c-myc HCC mice that had given a robust blood AFP response to iRGD one week earlier. In HCC mice injected with anti-NRP-1, there was no evidence for an iRGD-induced elevation of the blood AFP concentration (Fig. 3a), suggesting NRP-1-dependence of the iRGD-induced AFP transport in HCC mice.

The iRGD-induced increase in the blood AFP concentration correlated negatively with the basal (pre-injection) level of AFP in all four different HCC mouse models (Fig. 3b–e). HepG2 xenograft-bearing nude mice as well as TGFα/c-myc HCC mice with low basal blood AFP levels showed an iRGD-induced elevation of the blood AFP concentration, whereas animals with elevated basal blood AFP levels did not (Fig. 3f and g). These data suggest that iRGD-induced tumour-to-blood transport of AFP depends on the concentration gradient for AFP and that mice with HCC but still low blood AFP levels preferentially showed an iRGD-induced increase in the basal blood AFP levels. In mice with DEN-CCl_4_-induced HCC and low or high basal blood AFP levels, iRGD elicited an increase in the blood AFP concentration (Supplementary Fig. S6a), but the relative increase was stronger in animals with lower basal AFP levels (Supplementary Fig. S6b). Thus, the data obtained in this model are also consistent with the assumption that the iRGD-induced tumour-blood transport of AFP depends on the concentration gradient for AFP.

**Fig. 4:**
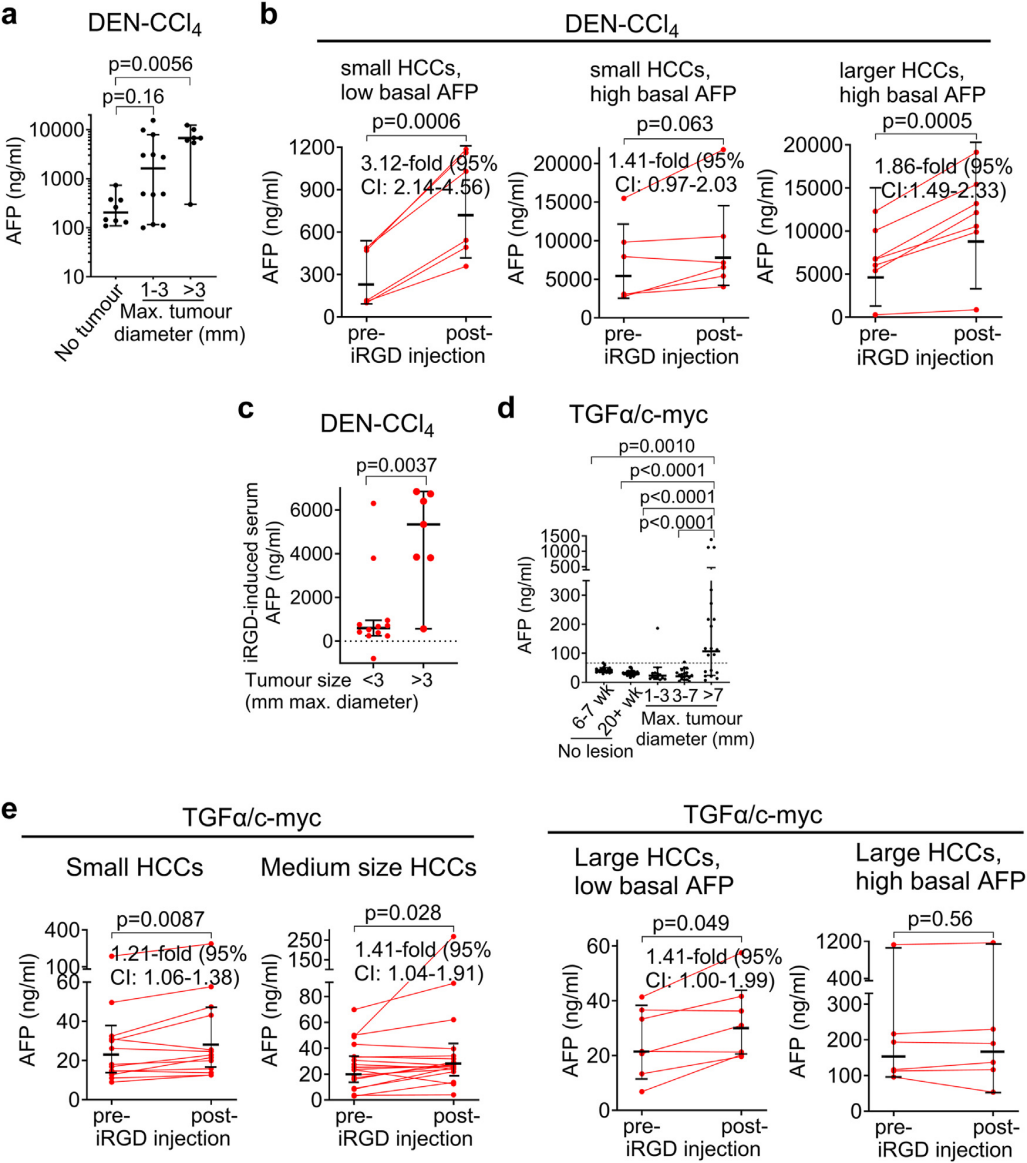
iRGD induces an increase of the blood AFP concentration in mice with small HCCs and low basal blood AFP levels. **(a)** Basal AFP levels in DEN-CCl_4_-treated mice prior to the tumour formation (week 15 of treatment) and in mice with small and larger HCCs. **(b)** iRGD increased blood AFP levels in DEN-CCl_4_-treated mice with liver fibrosis and small HCCs (tumours with maximal diameters of 1–3 mm according to MRI) and low blood AFP levels (>500 ng/ml, n = 6), and in animals with larger HCCs (tumours with maximal diameters >3 mm according to MRI) and low (>500 ng/ml), but not in mice with small HCCs and high basal AFP. **(c)** DEN-CCl_4_-treated mice with larger HCCs (>3 mm tumour diameter, n = 7) showed a stronger iRGD-induced increase of the blood AFP concentration than mice with smaller HCCs (<3 mm tumour diameter, n = 12). **(d)** Tumour size-dependence of the basal blood AFP level in TGFα/c-myc mice. AFP levels in TGFα/c-myc mice with no macroscopic liver tumours (6–7 weeks, n = 23, >20 weeks, n = 40) or tumours with maximal diameters of 1–3 mm (n = 13), 3–7 mm (n = 18) or >7 mm (n = 17) as graded by contrast-enhanced MRI. Dashed line: highest blood AFP concentration measured in a tumour-free animal. **(e)** iRGD responsiveness of blood AFP level in TGFα/c-myc mice with small (n = 13), medium (n = 18), or large liver tumours (n = 17). Fold increase of AFP with pre-injection level set to 1. **(a–e)** Lines and error bars represent geometric means (**b, c and e** top and left bottom) or medians (**a, c, and e**, right bottom) with 95% CI. **(a)** Kruskal–Wallis test with Dunn’s post-hoc multiple comparison; **(b and e)** paired t test or Wilcoxon matched-pairs signed-rank test (only e right, bottom) for log-transformed data; **(c)** Mann–Whitney U test. **(d)** One-way ANOVA for log-transformed data. **(b and e)** The indicated fold increase is the geometric mean ratio.

In DEN-CCl_4_-treated mice with HCCs >3 mm in diameter, AFP levels were clearly elevated, whereas no robust elevation of the blood AFP level were observed in animals with small HCCs (diameter 1–3 mm) (Fig. 4a). Remarkably, iRGD caused an elevation of the blood AFP concentration in mice with small HCCs and low basal AFP levels (<500 ng/ml) (Fig. 4b, left panel), i. e. in the range of tumour-free DEN-CCl_4_-treated animals (Fig. 4a), whereas iRGD was less effective in mice with small HCCs and high basal blood AFP levels (Fig. 4b, middle panel). In mice with basally elevated blood AFP levels and larger HCCs, iRGD nevertheless caused an increase of the blood AFP concentration (Fig. 4b, right panel). Further analysis of the tumour size dependence of iRGD-induced elevation of the blood AFP concentration revealed that iRGD elicited a much higher increase of the absolute blood AFP concentration in mice with larger HCCs as compared to mice with smaller HCCs (Fig. 4c), in agreement with the hypothesis that iRGD-induced tumour-blood transport of AFP depends on the amount of AFP in the tumours. Compatible data were obtained in TGFα/c-myc mice with HCC. TGFα/c-myc mice with small or medium size HCCs showed normal basal blood AFP levels; elevated basal blood AFP levels were almost exclusively seen in mice with large HCCs (Fig. 4d). iRGD treatment caused an elevation of the blood AFP level in a notable portion of TGFα/c-myc mice with small or medium size HCCs, i. e. in animals showing almost all normal blood AFP levels without iRGD (Fig. 4e). Histopathological examination revealed that the small liver lesions were already HCCs (Supplementary Fig. S7). In TGFα/c-myc mice with large HCCs, iRGD increased the blood AFP level only in animals with normal basal blood AFP levels, whereas iRGD was ineffective in animals with basally elevated blood AFP levels (Fig. 4e). Together, these data suggest that mice with small HCCs showed an iRGD-responsive blood AFP level unless the blood AFP concentration was already basally elevated.

### iRGD induces an increase in the blood concentration of autotaxin in mice with HCC and of the blood levels of PSA in mice with prostate cancer

**Fig. 5:**
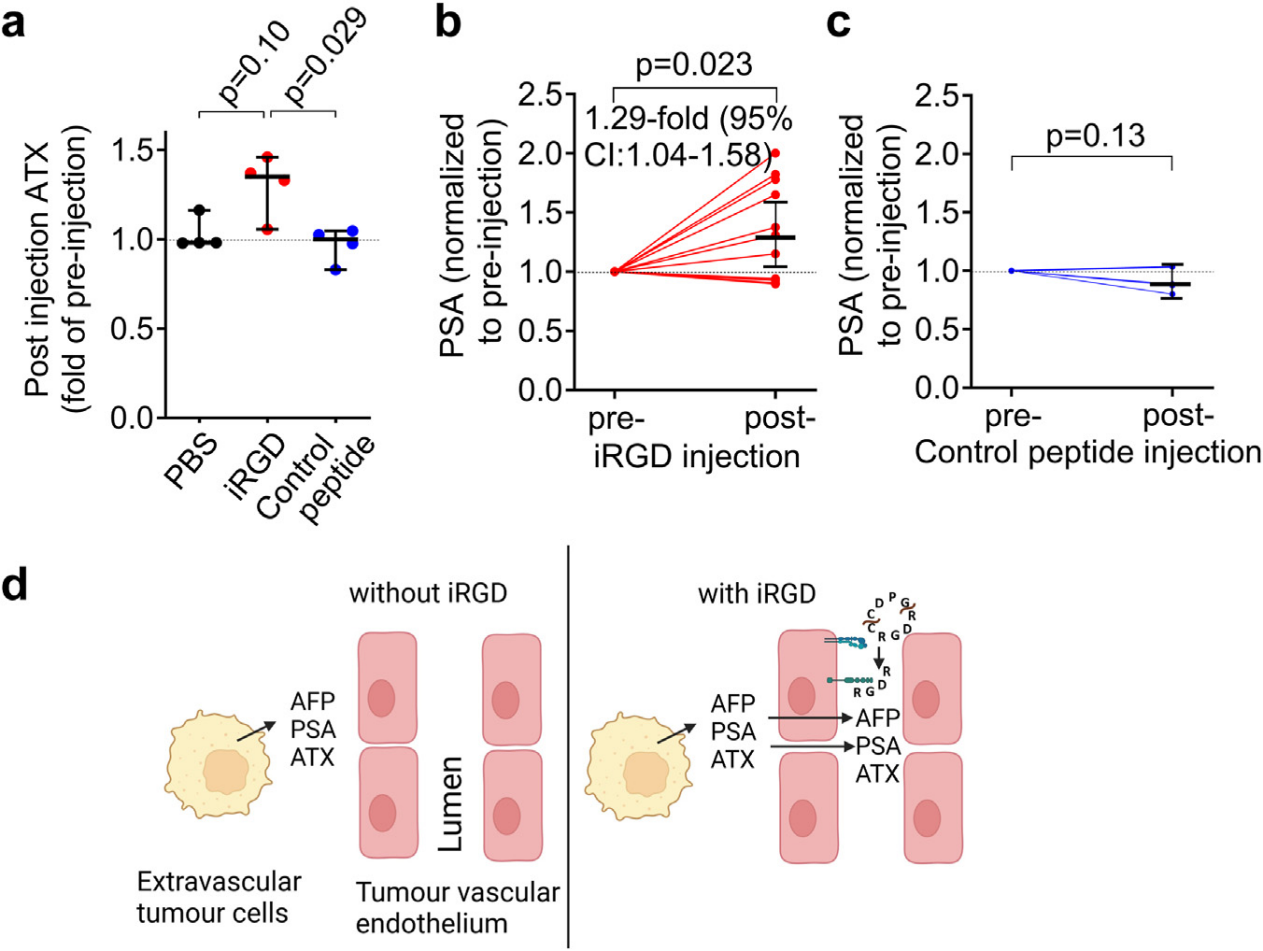
iRGD induces an increase in autotaxin (ATX) levels in HepG2-xenografted mice, and of PSA in LNCaP-xenografted mice. **(a)** Blood was drawn from HepG2 xenografted nude mice (tumours 0.6–1.5 cm in diameter) 5 min before and 90 min after the i.v. injection of iRGD. The sera were analysed for ATX content (n = 4 per group); lines and error bars represent medians and 95% CI. **(b and c)** Blood was drawn from mice xenografted with LNCaP prostate cancers (tumours 0.8–1.3 cm in diameter) 5 min before and 90 min after the i.v. injection of iRGD (n = 11) **(b)** or control peptide (n = 5) **(c)** and analysed for PSA content. Fold increase of AFP with pre-injection level set to 1. Lines and error bars represent medians **(a)** or geometric means **(b and c)** with 95% CI. **(a–c)** Statistical significance was calculated with Kruskal–Wallis test with Dunn’s multiple comparison post-hoc test **(a)** and one sample t test **(b and c)**. The indicated fold increase in **(b)** is the ratio of the geometric means with 95% CI. **(d)** Hypothetical model of the action of iRGD to induce a tumour-to-blood transport of tumour-secreted AFP, PSA and ATX. In the absence of iRGD, tumour-secreted AFP, PSA and ATX penetrate only slowly across the tumour endothelium (left panel), whereas in the presence of iRGD, the activation of NRP-1 by the CendR peptide generated from iRGD induces a paracellular and/or a transcellular transport across the vascular endothelium that transports AFP, PSA and ATX according to the tumour-blood concentration gradient (right panel). Created with BioRender.

If iRGD would also increase the transport of tumour-released proteins other than AFP into the blood, this may increase the portion of HCCs detectable by the iRGD manoeuvre and broaden the utility of iRGD in tumour detection. To address this issue, we examined the iRGD responsiveness of the blood levels of autotaxin, a secreted protein highly expressed in inflamed tissue and tumours.^44^ The measured blood autotaxin levels in nude mice xenografted with HepG2 tumours before and after the injection of iRGD were compatible with an elevated autotaxin level following injection of iRGD, whereas the RGD control peptide and vehicle had no detectable effect (Fig. 5a). These data suggest that iRGD increased tumour-to-blood transport of autotaxin in HCC mice.

To examine whether iRGD induces a tumour-to-blood transport of other tumour markers in another type of tumour, we investigated the effect of iRGD on blood levels of PSA in nude mice xenografted with LNCaP prostate cancer. Following injection of iRGD the blood levels of PSA were 1.3-fold higher as compared to the pre-injection level (Fig. 5b). No such change was observed in mice injected with the RGD control peptide (Fig. 5c). Collectively, these results suggest broader applicability of the iRGD effect on different tumour markers and tumour types (Fig. 5d).

### Discussion

Blood-based early tumour detection is critically limited by the amount of signal emitted from small/early-stage tumours, directly affecting the sensitivity.^9^, ^10^, ^11^, ^12^, ^13^ The specificity of blood-based tumour markers is reduced by signal release from non-malignant tissues. Here, we provide evidence that iRGD induces a transient tumour-to-blood transport in a highly tumour-specific fashion in tumour mice that may help to ameliorate these limitations of tumour markers and improve detection of early stage HCC and potentially other tumours.

Previous work has documented the ability of iRGD to enhance the permeability of the tumour vasculature and thereby increase tumour delivery of co-administered therapeutics in a highly tumour-specific fashion.^21^ The results presented here suggest that iRGD can also induce a transport of substances in the opposite direction, from the tumour interstitium across the vascular endothelium to the blood. The existence of this reverse transport was indicated by the appearance of increased tumour marker levels (AFP, PSA) and of the secretory protein autotaxin in the circulation as a result of iRGD administration to mice with HCC or prostate cancer, respectively (Fig. 5d). Similar to iRGD-induced tumour import, the iRGD-induced elevation of the blood AFP concentration was blocked by a neutralizing anti-NRP-1 antibody and the timing of the two effects showed similar rapid onsets, indicating a close relationship between the two transport processes. The lack of effect of iRGD/CendR peptide on AFP release from cultured HepG2 cells suggests that the iRGD-induced increase in the tumour-to-blood transport of AFP is not due to an increased secretion of AFP from the HCC cells into the tumour interstitium. The pronounced dependence of the iRGD-induced AFP transport on the basal blood concentration in all tested HCC mouse models suggests that this transport strongly depends on the concentration gradient of the substance to be transported between the tumour and the blood, a trait that also holds for VEGF-A-induced vascular leakage, which is also mediated by NRP-1.^45^

A remarkable finding of the present study is that iRGD elevated the tumour-blood transport of AFP in mice with small and medium-sized HCCs and normal/low basal blood AFP concentrations, suggesting that the iRGD-induced transport is already present in small HCCs and might thus be particularly useful to detect early-stage HCC that are difficult to diagnose in patients. Moreover, the apparent tumour specificity of the iRGD-induced reverse transport in tumours may provide a promising approach to overcome the difficulty of determining whether an increase of a potential circulating marker originates from a malignant tumour or from a non-malignant lesion that produces the marker, such as AFP in liver cancer vs. hepatitis. It should be noted that the efficacy of iRGD to induce an increase in the blood AFP concentration differed among the different HCC mouse models used. In DEN-CCl_4_ HCC mice the majority of animals showed an iRGD-induced increase of the blood AFP level, whereas in TGFα/c-myc mice this was only found in a portion of the HCC mice, probably due to AFP expression only in a portion of HCCs in this tumour model. The present study further shows that iRGD induces a tumour-blood transport of the important clinical tumour marker PSA in mice with prostate cancer, and of autotaxin, which has no tumour marker properties without iRGD, in HCC mice. Thus, it is possible that the iRGD-induced tumour-blood transport found in HCC is not limited to HCC and may be broadly present in malignant tumours, similar to the broad presence of iRGD-induced tumour import in different tumour types.^21^ The detection of an iRGD-triggered transport using sensitive and specific ELISAs allows reliable detection of small differences in the concentrations between the samples even at low concentrations, but does not disclose the spectrum of molecules undergoing iRGD-induced tumour-blood transport. The latter is difficult because only small amounts of blood can be obtained from living tumour mice, rendering the quantitative analysis e. g. of mutated ctDNA challenging.^11^

There is good evidence that the iRGD-induced tumour transport found in various tumour mice also occurs in human patients. α_v_ integrins and NRP-1 are also overexpressed in the vessels and stroma of human tumours, including HCC,^20^^,^^46^, ^47^, ^48^ and the iRGD-induced tumour uptake has been demonstrated in mice bearing patient-derived xenografts of pancreatic cancer,^26^ as well as *ex vivo* with human surgical tumour explants.^15^^,^^49^ The first-in-man study with iRGD in patients with pancreatic ductal adenocarcinoma was initiated based on the results in mouse tumour models representing a number different of tumour entities and shows that co-administered iRGD improves the therapeutic efficacy of various cancer therapeutics.^21^ It revealed favourable pharmacokinetics in humans and no signs of toxicity of iRGD as a monotherapy and in combination with nabpaclitaxel and gemcitabine and suggests that the use of iRGD led to an improved anti-tumour activity in these patients.^50^, ^51^, ^52^ Further clinical trials in patients with pancreatic cancer and other tumour entities are underway to test whether iRGD can improve the efficacy of standard therapy in this and other tumour entities.

A potential concern is that an iRGD-induced tumour-blood transport may enhance tumour metastasis, in particular as NRP-1 is known to have tumour-progressive effects such as stimulation of migration and angiogenesis.^53^ However, iRGD inhibits metastasis in tumour mice and migration of tumour cells *in vitro*,^54^^,^^55^ indicating that the administration of iRGD and the iRGD-induced tumour-to-blood transport might be safe.

Although the data showing the feasibility of iRGD-induced tumour-blood transport in HCC and prostate cancer are promising, several limitations exist in this study. First, iRGD has not yet been tested in patients with HCC. It will be crucial to evaluate whether the iRGD-induced tumour-blood transport occurs in patients with HCC and other tumours as revealed by changes in the blood levels of tumour-derived markers such as the clinical tumour markers AFP and PSA or emerging tumour markers. Second, the extent of elevation of tumour markers by the iRGD intervention may turn out to be too small to be clinically useful. Third, this study does not reveal the kinetics of tumour marker changes upon injection of iRGD and the optimal blood collection time. Fourth, iRGD may not increase the levels of tumour-derived markers in the blood when their levels are already strongly elevated without iRGD, as often observed in patients with advanced tumours.

In summary, we provide evidence that iRGD induces a tumour-to-blood transport without affecting normal tissues. This represents a promising approach to progress on the road towards improvement of early stage diagnosis of HCC and potentially other tumours by blood-based tumour markers.

## Contributors

CS, BK, DD, MvH, ACJ, OW, FF and AP designed the study and experiments. CS, BK, MvH, JHK and EHi performed animal experiments. CS, BK, DD, JHK, MvH, EHi, FF, AD, EHa, UC, PS, ACJ and AP conducted the *ex vivo* experiments and analysed the data. CS, DD and MvH had direct access to the mouse data. EHe and AP performed the statistical analysis. AP drafted the manuscript and wrote the paper, with important contributions of DD, SD, FF, BK, MvH, SZ, CS, JUM and OW. FF and AP supervised the whole project. All authors reviewed and edited the manuscript. CS and AP directly accessed and verified the data reported in the manuscript. All authors have read and approved the final version of the manuscript.

## Data sharing statement

Data generated by this study is available upon request to the corresponding author.

## Declaration of interests

OW: Personal fees from Amgen, Bayer, BMS, Celgene, Daiicgi Sankyo, Eisai, Incyte, Ipsen, Merck, MSD, Novartis, Pierre Fabre, Roche, Servier; honoraria for lectures and/or presentations from Amgen, AstraZeneca, Bayer, BMS, Eisai, Ipsen, MSD, Novartis, Roche, Zentiva; support for attending meetings and/or travel: Abbvie, AstraZeneca, Bayer, BMS, Gilead, Ipsen, Medac, Merck, Pierre Fabre, Roche. SZ: Consultancy and/or speaker’s bureau: Abbvie, BioMarin, Boehringer Ingelheim, Gilead, GSK, Ipsen, Madrigal, Merck/MSD, NovoNordisk, SoBi. JUM: Grants or contracts from any entity, AstraZeneca, consulting fees: AstraZeneca, Roche, Ipsen, Eisai; Payment or honoraria for lectures, presentations, speakers bureaus, manuscript writing or educational events: AstraZeneca, Roche, Ipsen, Eisai. The other authors declare no conflicts of interest.

## Appendix ASupplementary data

Supplementary Figs. S1–S7

Blotn
