# Supplementary material for: Tumour-specific activation of a tumour-blood transport improves the diagnostic accuracy of blood tumour markers in mice
Source: eBioMedicine. 2024 Jun 17;105:105178. doi: 10.1016/j.ebiom.2024.105178 (PMC11237870; doi:10.1016/j.ebiom.2024.105178)
Supplement: Supplementary Figs. S1–S7 [file mmc1.docx]

**Supplementary data**

**Tumour-specific activation of a tumour-blood transport improves the diagnostic accuracy of blood tumour markers in mice**

Christian Schmithals^1,+^, Bianca Kakoschky^1,+^, Dominic Denk^1,2,+^, Maike von Harten^1,+^, Jan Henrik Klug^1^, Edith Hintermann^3^ Anne Dropmann^4^, Eman Hamza^1,5^, Anne Claire Jacomin^2,6^, Jens U. Marquardt^7^, Stefan Zeuzem^1,8^, Peter Schirmacher^9^, Eva Herrmann^10^, Urs Christen^3^, Thomas J. Vogl^11^, Oliver Waidmann^1,12^, Steven Dooley^4^, Fabian Finkelmeier^1,2^, and Albrecht Piiper^1,2,8^

^+^Equally contributing authors

^1^Department of Medicine 1, University Hospital, Goethe University Frankfurt, Theodor-Stern-Kai 7, 60590 Frankfurt am Main Germany; ^2^Frankfurt Cancer Institute, Goethe University, Frankfurt am Main, Germany; ^3^Pharmazentrum Frankfurt / ZAFES, Goethe University Hospital, Frankfurt am Main, Germany; ^4^Molecular Hepatology-Alcohol Associated Diseases, Department of Medicine II, Medical Faculty Mannheim, University of Heidelberg, Germany; ^5^Suez University, Faculty of Science, Zoology Department, Suez, Egypt; ^6^Institute of Biochemistry II, Faculty of Medicine, Goethe University, Theodor-Stern-Kai 7, 60590 Frankfurt am Main, Germany; ^7^Department of Medicine I, University Medical Centre Schleswig-Holstein - Campus Lübeck, Lübeck, Germany; ^8^German Cancer Consortium (DKTK) and German Cancer Research Centre (DKFZ), Heidelberg, Germany; ^9^Institute of Pathology, University of Heidelberg, Germany; ^10^Goethe University Frankfurt, University Hospital, Institute of Biostatistics and Mathematical Modelling, Germany; ^11^Institute for Diagnostic and Interventional Radiology, University Hospital Frankfurt, Germany; ^12^Centrum für Hämatologie und Onkologie Bethanien, Frankfurt/Main, Germany

**Correspondence**: Albrecht Piiper, M. D., Ph. D., Department of Medicine 1, University of Frankfurt/M., Theodor-Stern-Kai 7, D-60590 Frankfurt, Phone: +49-6301-87667, FAX: +49-69-6301-87689, e-mail: [piiper@med.uni-frankfurt.de](mailto:piiper@med.uni-frankfurt.de)


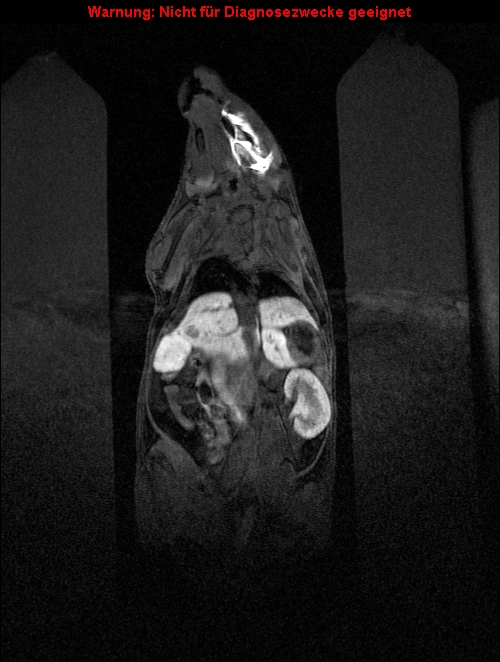

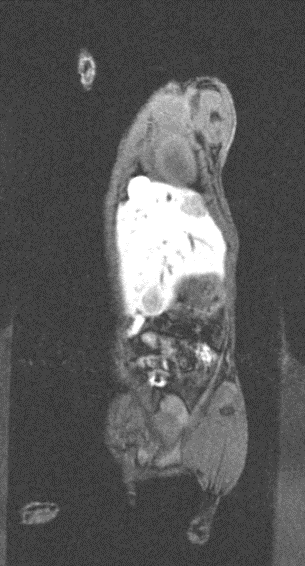


**Supplementary Fig. 1.** **Gd-EOB-DTPA-enhanced MRI of TGFα/c-myc mice with HCCs.** The blue arrows indicate HCCs.

**Supplementary Fig. 2. Intravenously injected iRGD increases blood AFP levels in HepG2 tumour xenograft-bearing mice.** **(a-d)** Measurement of the blood AFP levels before and after the intravenous injection of iRGD (n=18) (a and b), RGD control peptide (n=10) (c), and PBS (n=7) (c) into mice with HepG2 xenografts reveals a specific increase of the blood AFP concentration by iRGD. (d) iRGD had no influence on the blood levels of AST, ALT and LDH in HepG2 mice. Lines and error bars indicate geometric means (a-c) or medians (d) with 95% CI; (a and b): paired t test for log-transformed data; (c): unpaired t test. The indicated fold increase in (a and b) is the geometric mean ratio with 95% CI obtained from the mean differences of the log-transformed data.

**Supplementary Fig. 3. Histological evidence of liver fibrosis in Ad-2D6- or CCl_4_-treated mice and in Mdr2^-/-^ mice and treatment-induced elevation of the basal AFP levels.** Mice were treated with Ad-2D6 or CCl_4_ for four weeks; six month-old Mdr2^-/-^ mice developed liver fibrosis spontaneously. **(a)** Paraffin-embedded liver slices were stained with Haematoxylin/eosin (H&E) and Sirius red (n=5 per group). **(b and c)** Blood was drawn from the mice before the treatments and examined for AFP content. Lines and error bars indicate geometric means and 95% CI (b): n=14 per group; (C): n=6 per group; (b): unpaired t test with Welch correction for log-transformed data; (c): unpaired t test; bar = 100 µm.

**
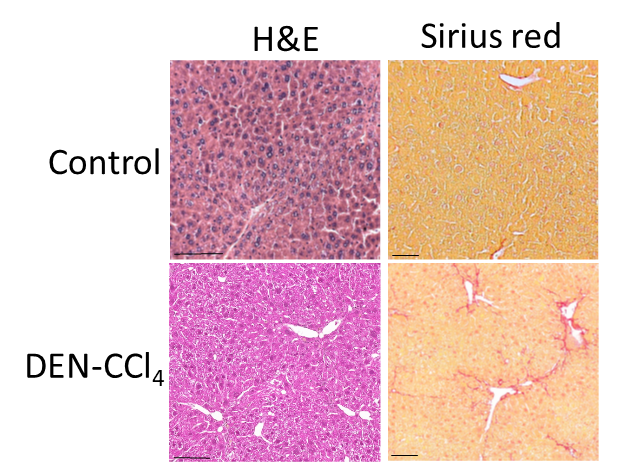
**

**Supplementary Fig. 4. Histological evidence of liver fibrosis in DEN-CCl_4_-treated mice.** Paraffin-embedded liver slices were stained with Haematoxylin/eosin (H&E) and Sirius red (n=4); bar = 200 µm.

**Supplementary Fig. 5. iRGD has no effect on AFP secretion from cultured HepG2 cells**. Data and error bars are means ± SD (n=3 per point of time).

**Supplementary Fig. 6. iRGD caused a higher fold increase in the blood AFP level in DEN-CCl_4_-HCC mice with low pre-injection blood AFP levels than in animals with high pre-injection blood AFP levels.** **(a)** Blood AFP levels before and after intravenous injection of iRGD from animals with low (<500 ng/ml) (n=7) or high (>500 ng/ml) (n=12) pre-injection AFP levels. **(b)** Fold increase in iRGD-induced elevation of the blood AFP level in DEN-CCl_4_-HCC mice. Lines and error bars represent geometric means and 95% CI. (a) paired t test for log-transformed data; (b) unpaired t test for log-transformed data. The indicated fold increase in (a) is the geometric mean ratio.


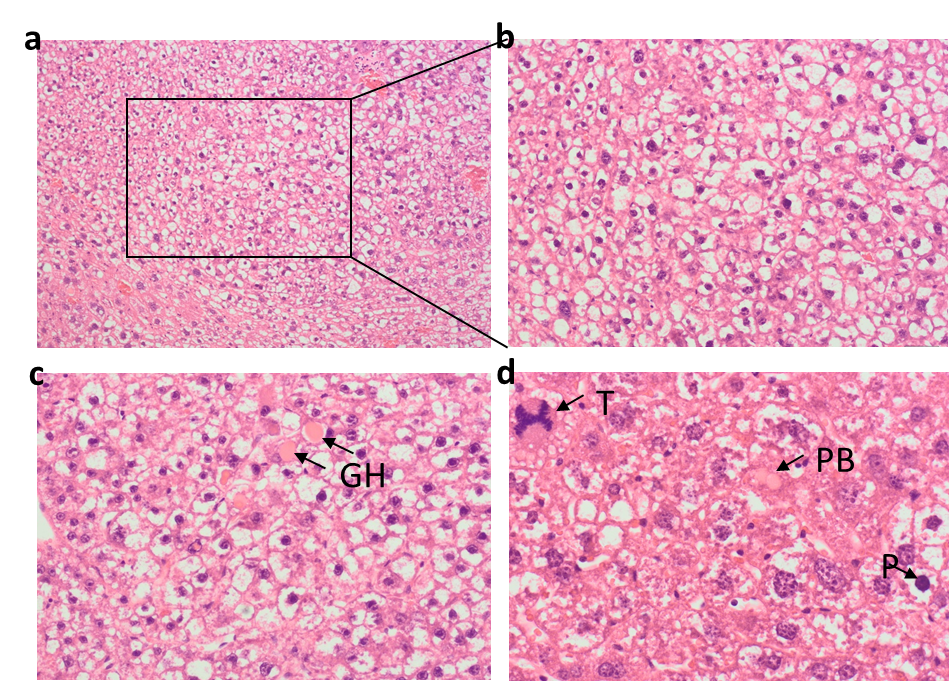


**Supplementary Fig. 7.** Examples of HE-stained small (1-3 mm in diameter) liver lesions of TGFα/c-myc mice: **(a** and **b)**: overview and detail of a HCC of clear cell type with pleomorphic, hyperchromatic nuclei, and focal occurrence of globular hyaline inclusion bodies (GH) (c); a tetrapolar mitosis (T), pale body inclusion (PB) and prophase nucleus (P) (d). Bar = 20 µm
