# Supplementary figures and images for: Tumour-specific activation of a tumour-blood transport improves the diagnostic accuracy of blood tumour markers in mice
Source: eBioMedicine. 2024 Jun 17;105:105178. doi: 10.1016/j.ebiom.2024.105178 (PMC11237870; doi:10.1016/j.ebiom.2024.105178)

## Slide 1
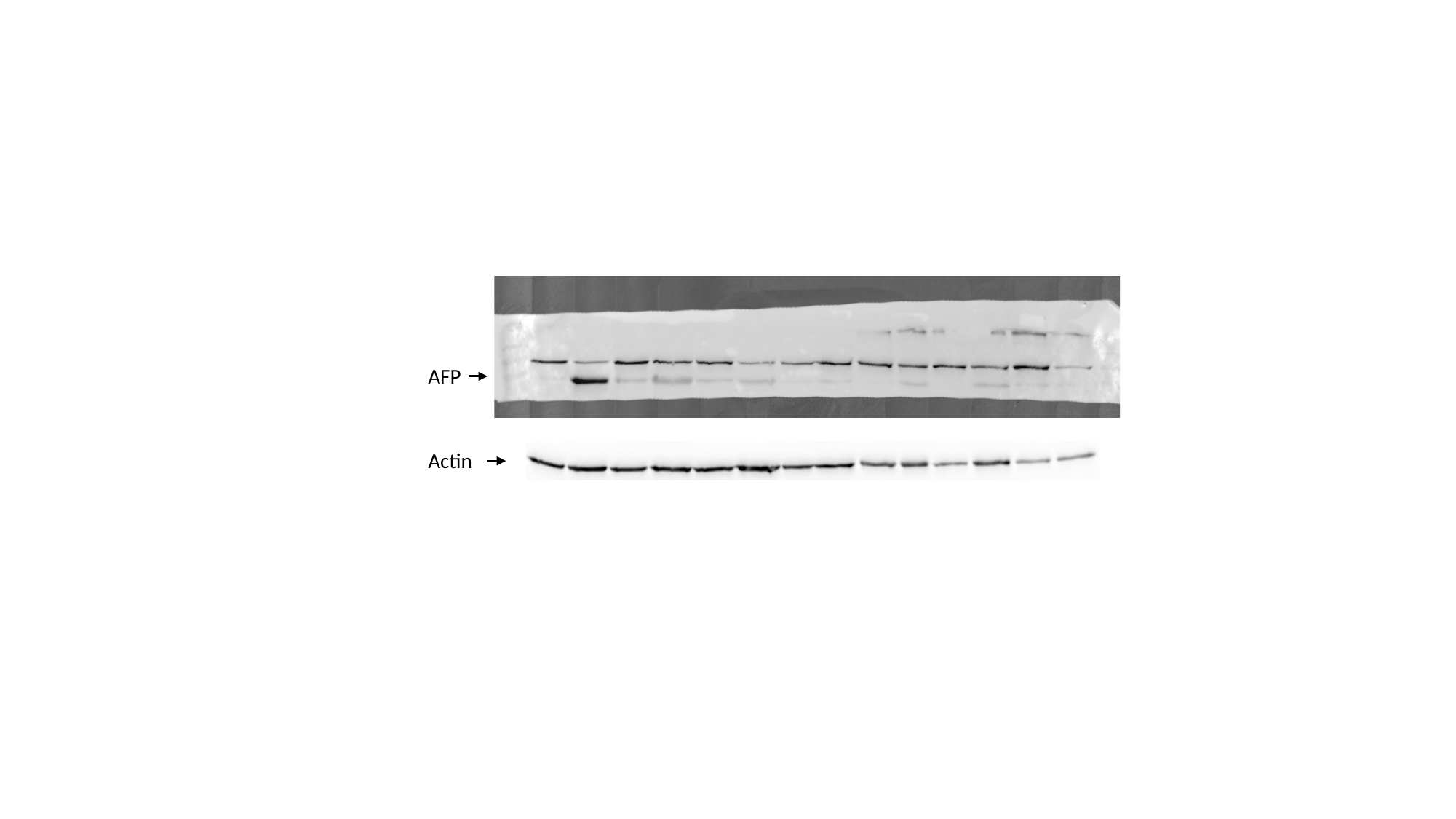

AFP
Actin

Supplement: Blotn [file mmc2.pptx]
